# Vaccination of boys or catch-up of girls above 11 years of age with the HPV-16/18 AS04-adjuvanted vaccine: where is the greatest benefit for cervical cancer prevention in Italy?

**DOI:** 10.1186/s12879-015-1067-9

**Published:** 2015-09-17

**Authors:** Paolo Bonanni, Giovanni Gabutti, Nadia Demarteau, Sara Boccalini, Giuseppe La Torre

**Affiliations:** Department of Health Sciences, University of Florence, Viale Morgagni 48, 50134 Florence, Italy; Department of Medical Sciences, University of Ferrara, Via Fossato di Mortara 64/b, 44121 Ferrara, Italy; Health Economics, GSK Vaccines, Avenue Fleming 20, 1300 Wavre, Belgium; Department of Public Health and Infectious Diseases, Sapienza University of Rome, Piazzale Aldo Moro 5, 00185 Rome, Italy

## Abstract

**Background:**

Since 2007, a Human Papillomavirus (HPV) vaccination programme against cervical cancer (CC) is implemented in Italy in 11-year-old girls. The extension of HPV vaccination to young adult women, or to 11-year-old boys could further reduce the CC burden, in the latter case from indirect effect on HPV transmission. The objective of the study was to compare the potential CC cases prevention from HPV-16/18 AS04-adjuvanted vaccination of adding catch-up targeting 15- or 25-year-old girls to the addition of boys vaccination in Italy.

The models assessing the impact of these alternative vaccination strategies are usually dynamic models requiring numerous input data. Simpler models could however provide some insight into this question, as reported in the current study.

**Methods:**

A published cohort model adapted to the Italian setting was used to estimate the potential CC reduction following different HPV vaccination strategies with a HPV-16/18 AS04-adjuvanted vaccine: vaccination of 11-year-old girls, female aged 15 or 25 years. The model assumed that the maximum benefit obtained from vaccinating boys equals the CC reduction that would result from immunisation of all non-vaccinated girls of the same age. Each cohort of 11-year-olds (either girls or boys) was assumed to include 281,000 individuals and a 70 % vaccination coverage was applied. Sensitivity analysis was performed by varying the vaccination coverage and the overlap in potential sexual contacts between vaccinated boys and girls of the same age-group.

**Results:**

Under base case, compared with the screening-only scenario, HPV vaccination of 11-year-old girls, 15-year-old females, 25-year-old females or 11-year-old boys, would prevent 1,146, 1,082, 788 or 491 CC cases respectively. HPV vaccination of boys could result in more CC cases prevented than adding a female catch-up only in scenarios with low vaccination coverage in the primary target cohort and when combined with small overlap between vaccinated boys and girls of the same age cohort.

**Conclusions:**

For a fixed limited additional budget allowing the inclusion of a single catch-up cohort, the extension of HPV vaccination to girls or young women instead of boys was estimated to maximise the number of CC cases prevented.

## Background

High-risk Human Papillomavirus (HPV) is the causal agent of cervical cancer (CC) [1, 2]. The persistent oncogenic HPV infection is the first step for the development of cervical precancerous lesions and, subsequently, of CC [3]. Oncogenic HPVs are also responsible for other anogenital cancers (primarily vagina, vulva, anus and penis) and their aetiological role, although with a lower attributable risk, has also been confirmed in head and neck cancers [4, 5].

According to data reported by the International Agency for Research on Cancer (IARC), CC is the fourth most common cancer in women in the world, and the seventh overall, with an estimated 527,624 new cases in 2012. In the same year, in the European Union, the IARC estimated 33,679 incident cases, 13,136 deaths, and the 5-year prevalence was 115,283 cases [6].

In Italy, CC was the fourth most frequent tumour in females aged 0–49 years in 2011 (6 % of overall incidence in the specific age-group). Although the incidence rate of CC showed a statistically significant reduction between 1998 and 2005, the mortality rate had a steady trend over the same period [7]. According to 9 Italian cancer registers, during the 2005–2009 period, 222 new CC cases were notified each year in Italy [7]. Other data from the Istituto Superiore di Sanità show that, for the 1998–2002 period, around 3,500 CC cases were diagnosed every year in Italy [8].

Two vaccines against HPV infection (a HPV-16/18 AS04-adjuvanted and a HPV-6/11/16/18 vaccine) are currently available in Italy and are used for the prevention of CC. Both vaccines have confirmed ability to reduce infection and precancerous lesions caused by HPV-vaccine types in a naïve girls population [9, 10]. The HPV-16/18 AS04-adjuvanted vaccine has also shown cross-protective efficacy against 4 oncogenic non-vaccine HPV types (HPV-33/31/45/51), possibly due to the presence of the AS04 Adjuvant System in the vaccine formulation, which enhances the overall immune response [11] as further confirmed by the 93 % overall efficacy shown against cervical intraepithelial neoplasia (CIN) grade 3+ lesions at year 4 of follow-up irrespective of HPV subtype [12]. The HPV-6/11/16/18 vaccine also has shown cross-protection versus HPV-31 although to a lesser extent [13, 14]. In Europe, the HPV-6/11/16/18 vaccine was granted an indication for boys vaccination in 2014 [15]. To date, the HPV-16/18 AS04-adjuvanted vaccine has no indication for boys; however, a clinical study has demonstrated immunologic response among boys similar to the one observed among girls [16, 17].

In Italy, HPV vaccination is actively offered free of charge to 11-year-old girls since 2007–2008 (depending on the region). In addition, some Italian regions have extended active offer to older female age-groups: 3 regions to girls aged 15 years, 4 regions to girls aged 16 years, 3 regions to young women aged 18 years and 1 region to young women aged 25 years (adopting a simultaneous 4-cohort strategy). In some regions, free-of-charge immunisation is maintained for some years after including an age cohort as target, and a co-payment system is foreseen in the other cohorts not included in the target age-cohort. In 2007, the coverage objective of the universal HPV immunisation programme was >95 % with 3 vaccine doses in 11-year-old girls, within 5 years from start. In the new Italian National Vaccination Plan (2012–2014), the coverage target was modified because of the difficulty in the achievement of the initial objective, with an adjusted 3-dose coverage of ≥70, ≥80 and ≥95 % in the 2001, 2002, and 2003 birth cohorts, respectively. At the end of 2012, a 70 % vaccination coverage with 3 doses of HPV vaccine was achieved in only 12, 10 and 8 Italian regions for the 1997, 1998 and 1999 birth cohorts respectively. Therefore, 5 years after the start of the immunisation programme against HPV in Italy, the vaccination coverage in the target population did not show the expected increase [18–20].

A larger impact of HPV vaccination could be reached by improving coverage among targeted girls, but alternative or additional approaches could also be sought. The addition of girls catch-up age-groups could be of benefit [21] but questions remain about the benefit of vaccinating boys [22].

Health authorities are currently questioning how the adoption of catch-up programmes in older age-cohorts of girls may compare with vaccination of boys, in addition to the efforts to increase vaccination coverage in the primary target group, in order to maximise CC prevention. As reported by the European Centre for Disease Prevention and Control (ECDC) in its last document ‘*Introduction of HPV vaccines in European Union countries*–*an update*’, the decisions to recommend the vaccine for boys and/or men depend on the epidemiology of HPV-related diseases in a specific country, the cost-effectiveness, and the affordability of the vaccine [23]. Therefore, the evaluation of the expected clinical outcomes resulting from the adoption of different vaccination scenarios involving “girls only” or “boys and girls” could help answer that question. Most models addressing this question are dynamic models requiring many input data limiting their adaptations to a few country settings, but also reducing transparency, hence making the evaluation of the model challenging [22].

Based on a simplified model, the objective of our analysis was to compare the potential additional number of CC cases avoided using the HPV-16/18 AS04-adjuvanted vaccine through different catch-up vaccination scenarios of older girls, or the vaccination of boys added to the vaccination of a first cohort of girls in Italy.

## Methods

This analysis uses the output of a published Markov model adapted to the Italian setting to estimate the CC reduction expected from different catch-up scenarios added to the primary target vaccination (15 or 25 years of age to cover the range of girls included in catch-up scenarios [24]), as well as the maximum potential effect of adding the vaccination of boys to the vaccination of girls (11 years of age) in terms of number of CC prevented at the country level, the ultimate aim of HPV vaccination [25, 26].

### Model description and input data

The analyses were conducted using an existing lifetime Markov cohort model developed in Microsoft Excel and adapted to the Italian setting [25]. The model has a 1-year cycle over 95 cycles (lifetime) to capture the entire potential effect of the vaccine. It reproduces the natural history of oncogenic HPV as well as the effect of screening and vaccination with 12 health states (healthy, oncogenic HPV infection, CIN1, CIN2/3, persistent CIN2/3, CC, CC cured, CC deaths, other deaths, CIN1 detected by screening, CIN2/3 detected by screening and persistent CIN2/3 detected by screening). The transition probabilities used to reproduce the progression and regression from healthy, to HPV infection, to lesion were retrieved from the literature (Table 1). Given the severity of the disease, limited data are available on the entire natural history of HPV disease. Lesions are treated upon detection to prevent further progression of the disease. No single study therefore reports all progression and regression rates needed to populate the model. Hence, multiple sources had to be used as generally used for similar model calibration [27–31]. Screening allows detecting lesions that can then be treated and potentially cured. Vaccination was assumed to reduce the transition from healthy to oncogenic HPV infection (hence reducing all subsequent HPV-related lesions) as of the age at vaccination. Therefore, the model assumes no effect of the vaccine on pre-existing infections or lesions that will continue to evolve according to the natural history. This allows estimating the effect of vaccinating girls after sexual debut [26].

**Table 1:** Input data in the mathematical model

	Parameter	Value	References
Vaccination	Cohort size	281,000	[53]
	Global vaccine efficacy CC (proxy CIN3+)	93 %	[12]
	Global vaccine efficacy CIN2/3 (proxy CIN2+)	65 %	[12]
	Global vaccine efficacy CIN1 (proxy CIN1+)	50 %	[12]
	Age at vaccination	11, 15 or 25 years	Assumed
	Vaccine waning	None	Assumed
Screening	Screening age range	25 to 64 years	[32]
	Screening interval	every 3 years	[32]
	Percentage screened	65 %	[32]
	Percentage never screened	35 %	[32]
	Cytology sensitivity	58–61 %	[54]
	Compliance to CIN 1 treatment	37 %	[32], expert opinion
	Compliance to CIN 2/3 treatment	100 %	[32], expert opinion
	Efficacy of CIN treatment	90 %	[32], expert opinion
Transition Probabilities	Healthy to HPV	0.07	[55]
	HPV to CIN 1	0.05	[56]
	CIN 1 to CIN 2/3	0.09	[57–59]
	CIN 2/3 to persistent CIN 2/3	0.11	[57, 59]
	HPV clearance to healthy	0.45	[60–64]
	CIN 1 clearance	0.24	[57–59]
	CIN 2/3 clearance	0.23	[57, 59]
	Persistent CIN 2/3 to CC	0–0.06	Calibrated

*CC* cervical cancer, *CIN* cervical intraepithelial neoplasia, *HPV* Human Papillomavirus

The vaccine effect is based on the expected effectiveness on CC. The prevention of CIN1 and CIN2/3 is however expected to be lower than the prevention of CC. The number of CIN1 and CIN2/3 post-vaccination were therefore adjusted according to the difference between the effectiveness on CC and the respective effectiveness on CIN1 and CIN2/3. Vaccine efficacy observed at year 4 of follow-up on the lesion irrespective of HPV types in the HPV-16/18 AS04-adjuvanted vaccine PATRICIA trial (NCT00122681) was used as a proxy of vaccine effectiveness at all ages (Table 1). It was therefore assumed that this observed efficacy applies to the Italian setting. A lifetime protection of the vaccination was assumed. The size of the 11-year-old girls cohort modelled was set at 281,000, based on the current demography in Italy (Table 1). The same size per age was assumed for a cohort of boys. All input data and assumptions included in the model are shown in Table 1.

### Model output

The incidence of CC cases over the lifetime of the cohort associated with different vaccination strategies was calculated by the model under 4 different scenarios:

- CC screening only
- CC screening and 100 % vaccination of females at the age of 11 years (primary vaccination target)
- CC screening and 100 % vaccination of females at the age of 15 years (15-year-old catch-up cohort)
- CC screening and 100 % vaccination of females at the age of 25 years (25-year-old catch-up cohort).

CC screening was assumed to take place every 3 years between the age of 25 and 64 years, and to cover 65 % of the female population [32]. These scenarios were used to estimate the lifetime number of CC cases remaining under the different vaccination coverage by calculating a weighted average of expected lifetime CC for strategies with and without vaccination (i.e. for a 30 % coverage, the lifetime CC were estimated by adding 70 % without vaccination and 30 % with vaccination). In the base case evaluation, HPV immunisation was assumed to cover 70 % of the vaccinated cohort.

### Estimation of the impact of vaccinating 11-year-old boys

The effect of boys vaccination, investigated in our evaluation, is estimated indirectly. The assumption taken is that the maximum benefit of boys vaccination would be to prevent the cervical cancers that are not already directly prevented by the girls vaccination. The impact of boys is therefore not derived from a potential efficacy on boys but well on the maximum expected impact on girls.

**Fig. 1:**
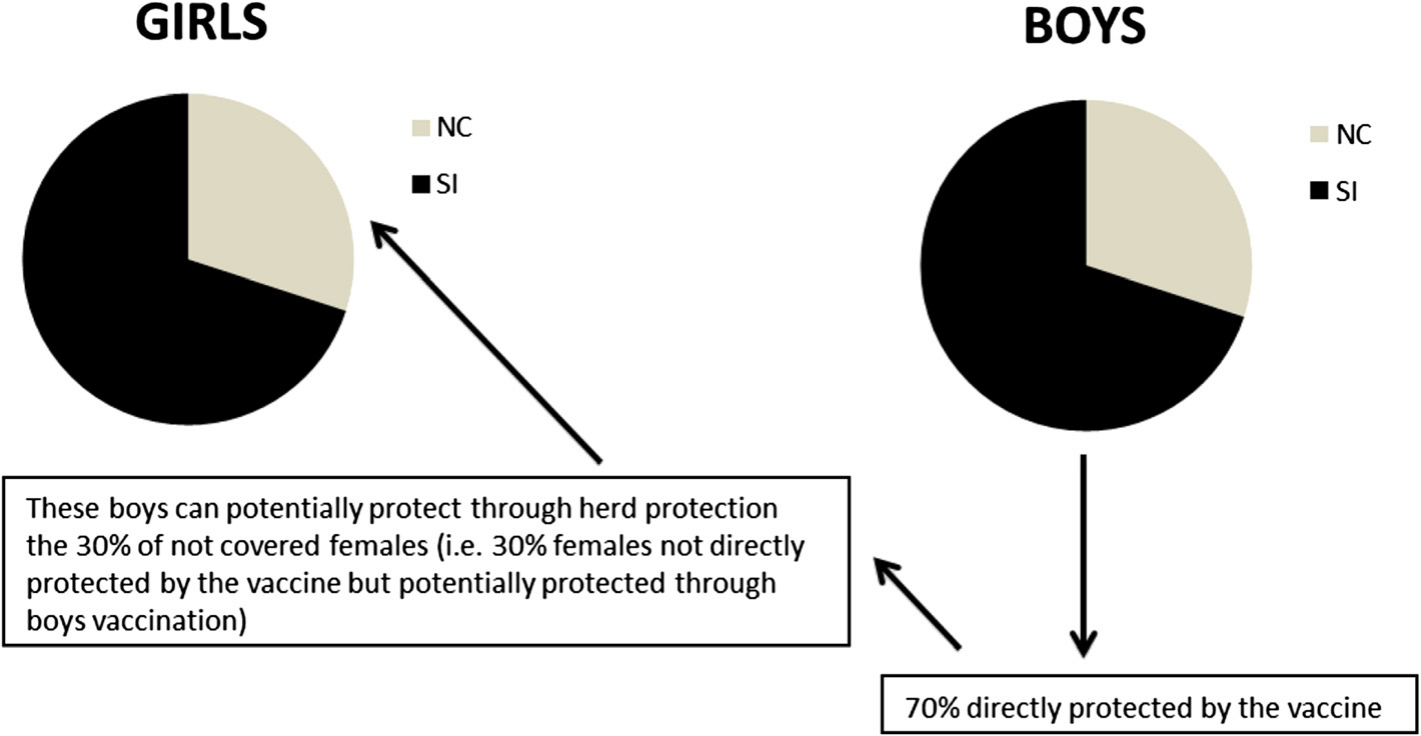
Schematic vaccination pattern investigated for a 11-year-old girls or boys cohort and resulting outcomes. Legend: *NC* not covered; 30 % of the cohort not directly protected by the vaccine, *SI* successfully immunised; 70 % of subjects directly protected by the vaccine

To estimate the potential CC reduction associated with vaccinating a 11-year-old boys cohort, it was assumed that the resulting maximum reduction in CC cases would correspond to the maximum expected reduction in CC cases of non-vaccinated 11-year-old girls as if they were all vaccinated. The reduction in the CC incidence from a 11-year-old boys vaccination, therefore, corresponds to the difference between the number of CC cases avoided under a 100 % vaccination coverage of a 11-year-old girls cohort and the number effectively prevented under the real vaccination coverage (i.e. 70 %). It was therefore assumed that the vaccination of boys would only lead to reduction in CC cases in the same age-group of females. This is schematically presented in Fig. 1.

### Sensitivity analysis

A one-way sensitivity analysis was conducted on the lifetime number of CC prevented under different vaccination coverage of both boys and girls. Two-way sensitivity analysis on lifetime CC incidence, accounting for a change in the vaccination coverage and the vaccination mismatch between vaccinated boys and girls was also performed. Specific sensitivity analyses of the duration of protection and cross-protection were also investigated.

#### Vaccination coverage among the catch-up cohorts

The vaccination coverage among the different scenarios was varied from 20 to 100 %. Under each scenario and coverage rate, the number of CC potentially prevented was estimated.

#### Vaccination mismatch between vaccinated boys and girls

The vaccination of boys can result in CC prevented only if vaccination of boys allows preventing CC among non-vaccinated girls. The mismatch factor allows taking into account the portion of the non-vaccinated girls protected by boys vaccination. It was ranged from 100 %, corresponding to the maximum benefit (i.e. the boys vaccination protects all non-vaccinated females), to 0 %, corresponding to the minimum benefit (i.e. the boys vaccination does not protect any of the non-vaccinated females as a result of exclusive interaction with vaccinated women).

#### Vaccine cross-protection and duration of protection

The impact of a reduced vaccine cross-protection or duration of protection on the results was investigated. The vaccination coverage in a girls catch-up programme below which the boys vaccination strategy (assuming maximum expected benefit from boys vaccination) is modelled to lead to more CC prevented than the addition of a catch-up programme was estimated for different vaccine efficacy and vaccine duration of protection. Vaccine efficacy was ranged using the observed 95 % confidence interval around average vaccine efficacy, irrespective of HPV type. The duration of protection was set at 10, 20 and 30 years assuming no booster.

## Results

### Model output

**Fig. 2:**
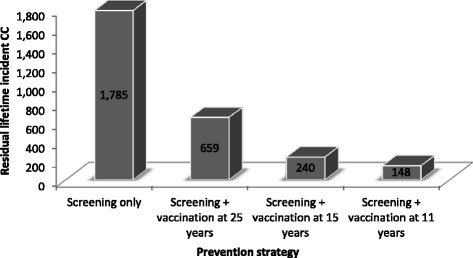
Lifetime incident cervical cancer cases per each cohort of 281,000 females under different prevention strategies targeting females (screening: 65 % screened every 3 years from 25 to 64; vaccination: 100 % vaccination coverage) used as input to the assessment model. Legend: *CC* cervical cancer

The remaining lifetime incidence of CC cases with or without the adoption of a HPV vaccination programme among females, in each analysed female cohort, as predicted by the model for a 100 % vaccination coverage, is presented in Fig. 2. These results are used as input data to the assessment model allowing the estimation of the CC cases under the different vaccination coverage and the maximum impact of boys vaccination.

HPV vaccination can only prevent incident infections. It is assumed to have no effect on the natural history of infections acquired before vaccination. As a result, the number of lifetime CC cases decreases between the scenario “screening only”, to vaccination at ages of 25, 15 and 11 years. The lifetime incident CC cases ranges from 1,785 CC for screening only to 148 CC for a 11-year-old girls vaccination, per cohort.

### Base case results

The number of CC cases prevented under the base case vaccination strategies (primary vaccination, catch-up cohorts and boys vaccination) is presented per cohort in Table 2.

**Table 2:** Cervical cancer averted under the base case vaccination strategy for each targeted cohort separately

Vaccination strategies	Cervical cancer prevented vs. screening only
*Primary vaccination*	
70 % 11-year-old girls vaccinated	1,146
*Catch-up programmes*	
70 % 15-year-old girls vaccinated	1,082
70 % 25-year-old women vaccinated	788
*Boys vaccination (maximum potential benefit among 11-year-olds)*	
70 % 11-year-old boys vaccinated	491

The maximum reduction of incident CC cases is obtained with the vaccination of the primary cohort (11-year-old girls), followed by a catch-up of a cohort of 15-year-old girls, then a catch-up of 25-year-old women. The vaccination of boys is expected to lead to the smallest reduction of CC.

### Sensitivity analysis

**Fig. 3:**
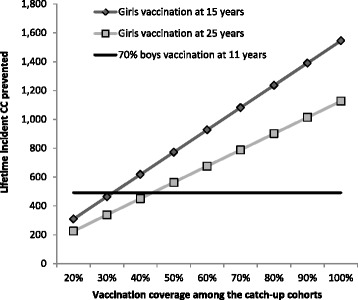
One-way sensitivity analysis on the lifetime incident cervical cancers prevented for a range of vaccination coverage for the catch-up cohorts. Legend: *CC* cervical cancer

**Fig. 4:**
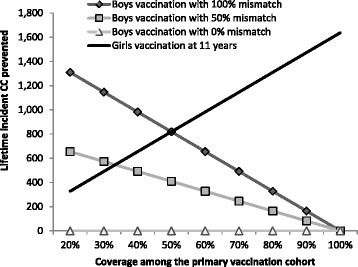
Two-way sensitivity analysis on the lifetime incident cervical cancers prevented for a range of vaccination coverage for the primary cohort and mismatch factor. Legend: *CC* cervical cancer

Results of the one-way sensitivity analysis on the lifetime incidence of CC cases prevented are presented in Fig. 3 and the two-way sensitivity analysis in Fig. 4.

Figure 3 reports the lifetime incident CC cases prevented for the catch-up vaccination scenarios and the vaccination of the 11-year-old boys for varying vaccination coverage for the catch-up cohorts, while assuming 70 % of the 11-year-old girls are covered. The vaccination of boys is predicted to result in more CC cases prevented than a catch-up cohort only for a vaccination coverage lower than 31 % for a catch-up of 15-year-old girls, and lower than 43 % for a catch-up of 25-year-old women.

Figure 4 reports the results of the two-way sensitivity analysis on the lifetime incidence of CC cases prevented among the 11-year-old cohort for both boys and girls strategies under different vaccination coverage for the 11-year-old girls or boys, and 3 scenarios (100, 50 and 0 %) for the mismatch between the vaccinated boys and girls.

The mismatch significantly impacts on the number of CC cases potentially prevented by the adoption of boys vaccination. Boys immunisation could lead to larger benefits for a vaccination coverage among girls lower than 50 % with a 100 % mismatch and 34 % with a 50 % mismatch. A 0 % mismatch could result, however, in no additional CC prevented from boys vaccination.

The vaccination coverage in a catch-up programme at which boys vaccination would lead to more CC prevented than the addition of a catch-up programme is reported in Table 3 under different vaccine profiles regarding the overall efficacy or the duration of protection.

**Table 3:** One-way sensitivity analysis on the vaccination coverage below which a full boys vaccination leads to more cervical cancer cases prevented than a catch-up vaccination

	Vaccination coverage	
Parameter varied in the sensitivity analysis	15-year-old girls catch-up	25-year-old women catch-up
	% coverage (*N* girls)	% coverage (*N* girls)
Base case (VE 93 %, lifetime duration of protection)	31 % (87,110)	43 % (120,830)
VE = 79 %	31 % (87,110)	43 % (120,830)
VE = 99 %	31 % (87,110)	43 % (120,830)
Vaccine duration of protection = 10 years	27 % (75,870)	30 % (84,300)
Vaccine duration of protection = 20 years	29 % (81,490)	34 % (95,540)
Vaccine duration of protection = 30 years	30 % (84,300)	39 % (109,590)

*VE* Vaccine efficacy

## Discussion

Achieving high HPV vaccination coverage in the primary target group (11-year-old girls) is the top priority concerning HPV immunisation programmes in all developed countries for a number of reasons: vaccination before the sexual debut maximises the impact of immunisation; pre-adolescents show an excellent immunological response to HPV vaccines; therefore, also from an economic point of view, 11-year-old girls immunisation is the most effective option. However, in Italy, HPV vaccination coverage did not reach the foreseen 95 % coverage objective yet.

A better impact of HPV vaccination on the incidence of CC cases could be achieved with the addition of a cohort of vaccinated females in the age range of 15–25 years. The adoption of a multi-cohort strategy might protect age-groups that are entering or are already at risk of incident HPV infection. This kind of preventive intervention (active and free-of-charge offer to secondary age-groups) is already adopted in some Italian regions (Valle d’Aosta, Piemonte, Liguria, Trentino-Alto Adige, Friuli-Venezia Giulia, Toscana, Marche, Basilicata and Puglia).

Another possible prevention strategy could be the expansion of HPV vaccination to 11-year-old boys, in addition to the primary girls vaccination. In fact, some Italian regions have already introduced active free-of-charge HPV vaccination of males (Veneto, Liguria, Friuli-Venezia Giulia, Puglia, Sicilia and Basilicata).

Recently, some other countries, like USA and Canada, also included HPV vaccination of boys in immunisation programmes. As a matter of fact, the American Centers for Disease Control and Prevention (CDC) recommended that both young boys and girls should be vaccinated against HPV because of disappointing coverage rate in girls reached in the last years (49 % of adolescent girls received at least the first of the 3 doses of HPV vaccine in 2010). Vaccination of males would likely reduce HPV transmission, and resulting infection, disease, and cancers in females (through herd immunity) [33, 34]. However, very few adolescent males received the HPV vaccine during the first year following its recommendation for this gender in the USA. Therefore, in order to reach high vaccination coverage in boys, compliance of adolescent males and their parents should be improved [35, 36]. A widespread acceptance by recipients and parents is a key point for the successful implementation of the HPV vaccination programme. According to a systematic review, the current low coverage of HPV immunisation by active offer is due to: scarce knowledge of HPV and the HPV vaccines; high costs in countries where these costs are incurred by the recipient or his/her parents; perceived low efficacy of the vaccine; and alleged and real adverse events to HPV vaccines [37]. However, females perceive the greatest health benefit through HPV immunisation (avoidance of CC), with a better acceptance of vaccination. This perception of health gain is less evident in males, and the introduction of boys vaccination to coverage levels similar to those achieved in females might be very difficult to reach.

In many European countries, as in Italy, coverage rates of girls immunisation reached lower levels than expected, despite the relevant efforts made by health authorities [38]. The most effective strategy to prevent HPV-related morbidity could be universal immunisation as the inclusion of boys in the current HPV vaccination programme is likely to be beneficial to both sexes: boys immunisation, in addition to indirectly protecting cancer in girls, is directly effective in the prevention of HPV-related conditions in men (such as anal cancer). In addition, despite the clinical benefits of universal vaccination, the immunisation programmes including boys in the current HPV vaccination strategies are presently not considered a priority. From this point of view, the ECDC position expressed in the recent guidance related to the introduction of HPV vaccines in European Union countries (2012) suggests that universal immunisation programmes for boys seem to be too costly compared to the potential benefits [38]. These evaluations were based on the results of dynamic models.

We developed a simpler model to estimate the potential relative efficiency of immunisation on CC, comparing boys HPV vaccination with a catch-up HPV vaccination programme in older adolescents and young women. A series of assumptions had therefore to be made. The indirect efficacy of boys vaccination on CC is unknown. We did therefore make assumption by estimating that the maximum benefit of boys vaccination would result from prevention of CC among girls in the same age-group. However, boys vaccination could benefit younger or older girls or on the contrary protect a limited number of girls in the same age-group. The exact proportion of non-vaccinated girls that could be protected is therefore difficult to exactly measure. A potential weakness of our study is that it only focuses on the prevention of CC, which, however, represents the major HPV-related burden in cancer cases. Nevertheless, the approach of our model could be extended to other HPV-related diseases. In that case, additional assumptions would need to be taken into account for the potential direct and indirect protection afforded by immunisation from either boys or girls vaccination.

The results of our analysis suggest that the vaccination of an additional cohort of 15- or 25-year-old females in a catch-up programme would save a larger number of CC cases than an immunisation programme also for 11-year-old boys. The vaccination of boys with HPV vaccine was estimated to result in a larger CC reduction than a catch-up programme in young adult women only in a scenario of low vaccination coverage in the primary target, which is not currently the case in Italy. Therefore, in a setting with relatively high vaccination coverage among 11-year-old girls, adding a catch-up vaccination programme among older girls would prevent more CC cases than adding boys vaccination. The model also highlights the importance of overlap between vaccinated boys and girls: a 0 % mismatch between vaccinated boys and vaccinated girls corresponding to all vaccinated girls being in contact with the vaccinated boys only, could result in no additional benefit from boys vaccination. The maximum benefit from boys vaccination appears when 100 % mismatch exists between the two vaccinated groups. This corresponds to boys mixing with unvaccinated girls, hence all unvaccinated girls would indirectly be protected by boys vaccination.

In relative terms, these results are insensitive to the vaccine efficacy. Indeed, a change in the vaccine efficacy does impact the overall expected reduction in CC cases (CC directly prevented by the girls vaccination + the CC indirectly prevented by boys vaccination). The change in vaccine efficacy would therefore not modify the coverage in the catch-up programme under which the vaccination of boys would prevent more CC than the catch-up of girls. As a result, a lower vaccine efficacy would lead to a proportional decrease in both the boys and the catch-up vaccination.

From this modelling exercise, the adoption of boys vaccination could result in the prevention of more CC cases than adding catch-up female cohorts only if low vaccination coverage is reached in girls (catch-up or primary vaccination cohort). This should however imply a maintained high (non-overlapping) coverage in boys that would result in the maximum CC reduction modelled in this study. Nevertheless, if the benefit of vaccinating boys goes beyond CC prevention among the girls not covered in the primary cohort (important age mix with older non-vaccinated girls), the value of a boys vaccination could potentially be more important.

A potential limitation of our analysis could be the assumption of a lifelong duration of protection. However, in view of the effectiveness profile of the HPV-16/18 AS04-adjuvanted vaccine, and lacking any forecast on its possible decay, this assumption might be reasonable [39]. Nevertheless, the sensitivity analysis conducted on the vaccine duration of protection confirmed the results of the base case.

We also assumed that the maximum reduction in CC cases subsequent to the extension of HPV vaccination to 11-year-old boys would correspond to the maximum expected reduction in CC cases of non-vaccinated 11-year-old girls as if they were all vaccinated. Such assumption does not account for a possible more extended impact in case vaccinated males had frequent sexual contacts with older unvaccinated females. Nevertheless, such effect, although possible, would most probably be negligible compared to the overall impact on females of the same age.

The results of this analysis are based on a simplified evaluation compared with evaluation made with dynamic models. However, they correlate with data obtained from other previous studies based on more complex published dynamic models [22, 40–52]. These studies have all estimated a lower Incremental Cost-Effectiveness Ratio (ICER) for girls-only vaccination compared with girls-and-boys vaccination, the latter being not cost-effective in most cases. They also estimated that the vaccination of boys would become cost-effective for low vaccination coverage on the condition, for example, of a low vaccine price. The effect of vaccine price was however not assessed in the current evaluation. The advantage in our study is that it allows for adaptation to countries with less extensive input data than the ones needed for a dynamic model, and to be transparent about the model input and output. In the absence of the data and computer power needed to run a dynamic model, the approach used in the current evaluation provide an alternative for countries willing to compare alternative vaccination strategies.

## Conclusions

Our analysis shows that HPV vaccination of 11-year-old boys might have limited benefits on CC incidence. It would be relevant only if female HPV vaccination coverage related to primary immunisation target cohort is low. Increasing that female coverage would be a more efficient strategy than including male vaccination to reduce the overall health burden of CC in the population. Therefore, in Italy, where the HPV vaccination coverage of the primary target population (11-year-old girls) is about 69 % in the 1997 and 1998 birth cohorts, extending vaccination to more girls (females of the primary target cohort and/or older girls) instead of adding boys immunisation is predicted to maximise the number of CC cases prevented under a fixed limited additional budget.
